# Lesão de Hill-Sachs: Diagnóstico, classificação e tratamento

**DOI:** 10.1055/s-0045-1809525

**Published:** 2025-07-10

**Authors:** Marcel Jun Sugawara Tamaoki, Artur Yudi Utino, Renato Aroca Zan, Fabio Teruo Matsunaga, Nicola Archetti Netto

**Affiliations:** 1 Disciplina de Cirurgia da Mão e Membro Superior, Grupo de Ombro e Cotovelo, Departamento de Ortopedia e Traumatologia, Escola Paulista de Medicina, Universidade Federal de São Paulo, São Paulo, SP, Brasil

**Keywords:** fratura do úmero, instabilidade articular, luxação do ombro, procedimentos ortopédicos, humeral fractures, joint instability, orthopedic procedures, shoulder dislocation

## Abstract

A lesão de Hill-Sachs é uma condição frequentemente associada à instabilidade anterior do ombro, que desempenha um papel crucial nos resultados de seu tratamento. É caracterizada por uma fratura compressiva posterior na cabeça do úmero, que resulta do impacto contra a borda anterior da cavidade glenoidal durante um episódio de luxação. A compreensão aprofundada dessa lesão é essencial para embasar decisões clínicas e para a escolha do tratamento mais adequado. Com os avanços nas modalidades de imagem, como a ressonância magnética e a tomografia computadorizada, tornou-se possível identificar a presença da lesão com maior precisão e classificá-la de acordo com sua profundidade, localização e volume, o que possibilita uma avaliação mais detalhada do seu papel na instabilidade do ombro. Este artigo revisa as principais classificações, métodos de diagnóstico e opções de tratamento, com o objetivo de fornecer ao ortopedista uma visão abrangente e atualizada das estratégias que promovem melhores desfechos funcionais e minimizam o risco de recorrência da instabilidade.

## Abstract (en)

A lesão de Hill-Sachs é uma condição frequentemente associada à instabilidade anterior do ombro, que desempenha um papel crucial nos resultados de seu tratamento The Hill-Sachs lesion is a condition frequently associated with anterior shoulder instability, which plays a crucial role in the outcomes of its treatment. It is characterized by a posterior compressive fracture in the humeral head caused by an impingement against the anterior rim of the glenoid cavity during dislocation. A thorough understanding of this lesion is essential to support clinical decisions and choose the most appropriate treatment. Advances in imaging modalities, such as magnetic resonance imaging and computed tomography, enabled lesion identification with greater precision and its classification per depth, location, and volume, resulting in a detailed assessment of its role in shoulder instability. The present article reviews the main classifications, diagnostic methods, and treatment options to provide orthopedists with a comprehensive and updated view of the strategies to promote better functional outcomes and minimize the risk of instability recurrence.

## Introdução

Apesar de ser amplamente conhecida, há muito interesse na lesão de Hill-Sachs (HS), uma vez que ainda existe muita discussão acerca de como ela contribui para o desenvolvimento da instabilidade do ombro, bem como suas implicações no tratamento dos pacientes com essa condição, seja por meio de abordagem cirúrgica ou não operatória.

A primeira descrição da luxação do ombro remonta ao antigo Egito, em cerca 3000 AC, no papiro de Edwin Smith. Já a lesão encontrada na cabeça umeral foi mencionada pela primeira vez por Malgaigne em 1855. Em 1940, 2 radiologistas, Harold Arthur Hill e Maurice David Sachs, descreveram e nomearam a lesão, 1 2 que é definida como uma depressão da cabeça umeral na região posterolateral, e ocorre em associação com a luxação anterior do ombro. Essa fratura por compressão ocorre devido ao impacto do osso esponjoso da cabeça umeral contra a cortical anterior da cavidade glenoidal. 3 Essa lesão, associada ao comprometimento ósseo ou labial na face anterior da cavidade glenoidal, pode estar associada à instabilidade do ombro. 4

Atualmente, os dados indicam que a luxação do ombro acomete principalmente a população jovem e ativa, o que gera preocupação acerca das implicações socioeconômicas dessa condição. 5

A incidência de lesão de HS varia entre 40% e 90% nas luxações anteriores do ombro, sendo que, na luxação redicivante, pode chegar a até 100% dos casos. 3 4 6 É importante destacar que a lesão de HS raramente ocorre isoladamente, o que reforça o conceito de lesão bipolar (associada à lesão da cavidade glenoidal da escápula), que está presente em 63% das ocasiões. 7 8

O posicionamento do braço no momento da luxação é um dado relevante, uma vez que a localização e a inclinação da lesão de HS criada afetam a estabilidade do ombro. Se a luxação ocorrer com o ombro em abdução, ocasionará um maior risco de *engagement* (encaixe da lesão de HS na borda anterior da glenóide). 9 Lesões de HS mais extensas, especialmente as mais medializadas, 4 do mesmo modo, aumentam o risco de instabilidade devido à redução do contato da cabeça do úmero com a superfície articular da cavidade glenoidal.

## Histórico e Quadro Clínico

O paciente com a lesão de HS tipicamente apresenta queixa de instabilidade. Pode ter histórico associado de dor no ombro, que piora nas posições de abdução ou hiperextensão dessa articulação, 3 ou, ainda, sinais como crepitação e estalidos à movimentação. A possibilidade de uma nova luxação aumenta com o número de episódios e com o tamanho do defeito ósseo. O teste de apreensão positivo com graus menores de abdução ( *mid range* ) é mais associado a defeitos na cavidade glenoidal. Já essa positividade encontrada no final da abdução e rotação lateral ( *end range* ) atribui-se à presença de lesão de HS. 4

Até o momento, não há uma descrição de uma manobra propedêutica específica para avaliação da lesão de HS. O exame físico deve ser conduzido rotineiramente para avaliar a instabilidade do ombro, que inclui avaliação da frouxidão ligamentar generalizada (critérios de Beighton), realização dos testes do sulco, de Gagey, de apreensão, surpresa, e de recolocação. 10

Outros testes a serem realizados são o teste de hiperextensão e rotação interna (hyperextension–internal rotation [HERI] test) 11 e o teste de apreensão óssea. 12 Ainda há controvérsia na literatura sobre a sensação de instabilidade em graus menores de abdução (teste de apreensão óssea) em detectar perda óssea significativa na cavidade glenoidal ou no úmero (HS). 12 13 É importante a confirmação do achado de luxação/instabilidade no exame físico sob anestesia.

## Exames de Imagem

**Fig. 1:**
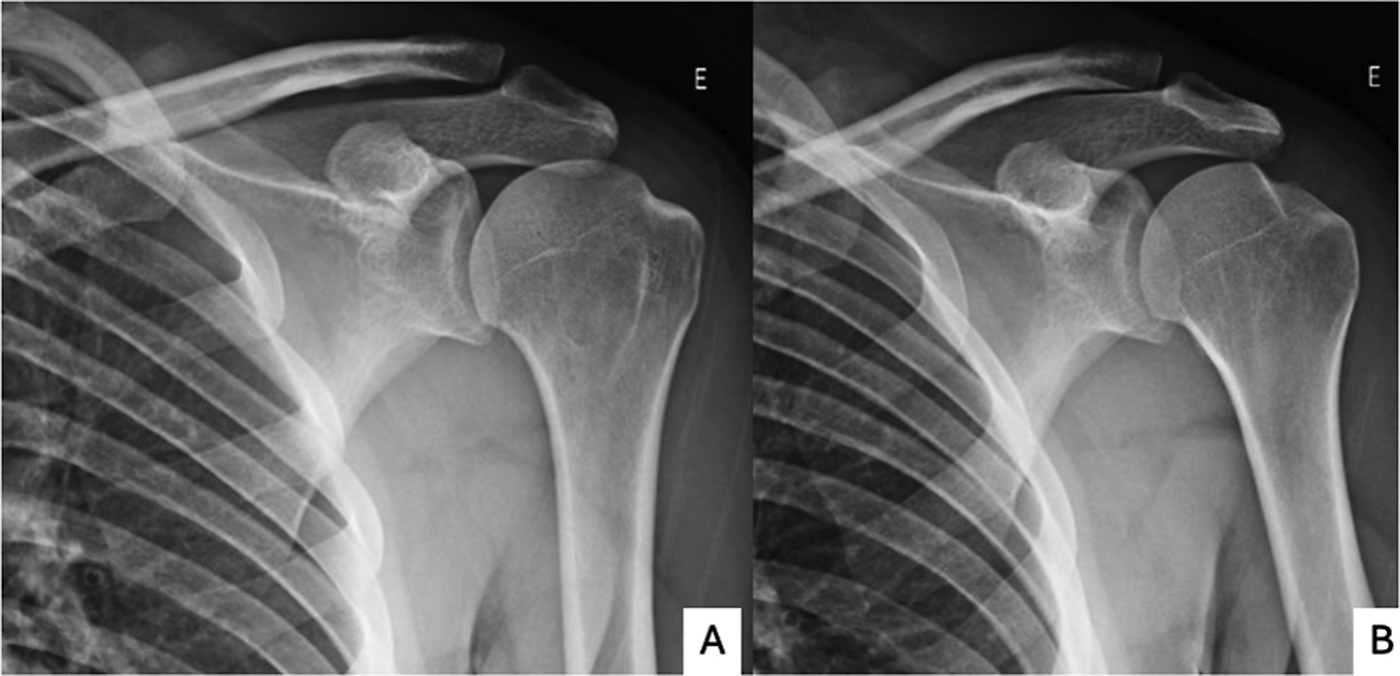
Radiografias do ombro com lesão de Hill-Sachs (HS) nas incidências anteroposterior (AP) verdadeira, AP, de perfil axilar e de perfil da escápula.

Radiografias do ombro nas incidências clássicas anteroposterior (AP), AP verdadeira, de perfil, de perfil da escápula e de perfil axilar são utilizadas de maneira rotineira na investigação inicial ( Fig. 1 ).

Na investigação radiográfica específica do defeito ósseo umeral da lesão de HS, podemos lançar mão das seguintes incidências radiográficas: 3

**Fig. 2:**
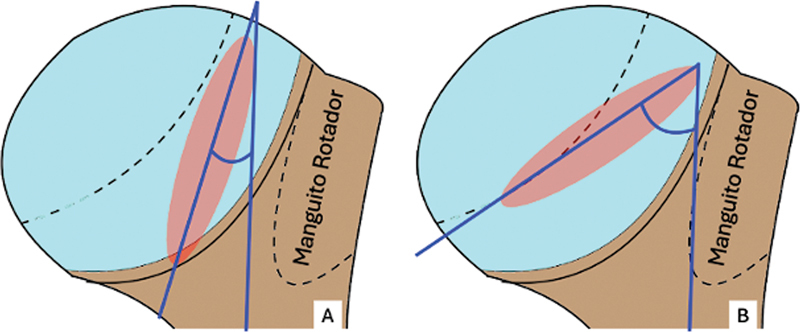
( **A** ) Radiografia AP com rotação lateral, que não evidencia a lesão de HS; ( **B** ) incidência AP com leve rotação medial, que evidencia a lesão de HS.

- *Stryker notch* : a rotação medial da cabeça umeral evidencia o defeito posterolateral.
- *Garth view* : incidência AP do ombro no plano da escápula, com 45° de inclinação caudal do raio.
- AP com rotação medial: pode evidenciar o tamanho, a profundidade e a orientação da lesão de HS na região posterolateral da cabeça umeral. 14 Muitas vezes, a incidência AP com rotação lateral não evidencia a lesão; consegue-se visualizar somente uma rarefação óssea medial ao tubérculo maior 1 15 ( Fig. 2 ).
- Didier modificada: paciente em decúbito ventral com o dorso da mão apoiado na crista ilíaca posterior e o cotovelo fletido, com raio angulado em 45° graus em relação ao solo, no sentido da cabeça umeral. 16

Radiografias apresentam baixa confiabilidade interobservador, e não são capazes de fornecer dados necessários suficientes para o planejamento pré-operatório, 17 uma vez que até 60% dos defeitos ósseos podem ser negligenciados nos casos em que há a análise somente por esse método.

**Fig. 3:**
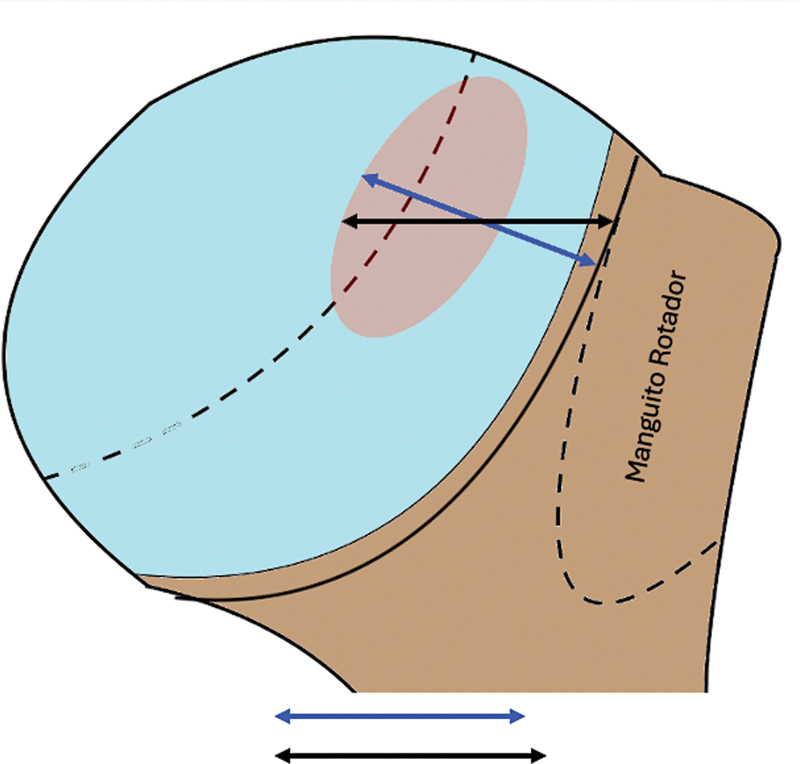
Imagens de tomografia computadorizada (TC) que evidenciam a lesão de HS. Imagens bidimensionais nos cortes axial, sagital e coronal. Imagens tridimensionais nas visões lateral e posterior.

A tomografia computadorizada (TC) e a ressonância magnética (RM) são métodos de imagem complementares ( Figs. 3 4 ), que apresentam maior sensibilidade do que as radiografias para detectar as lesões de HS. 14

**Fig. 4:**
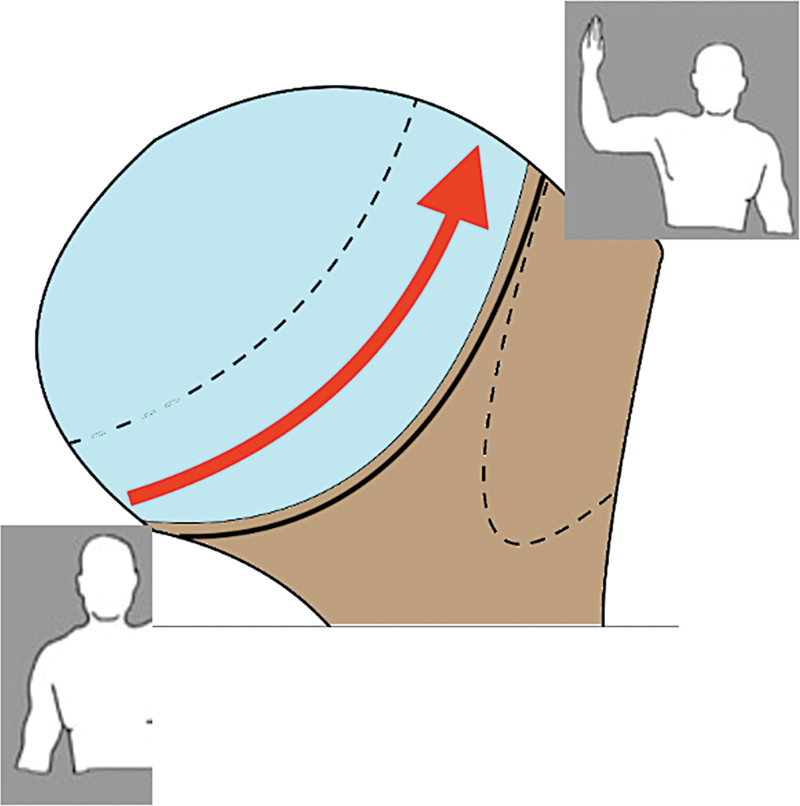
Imagens de ressonância magnética (RM) em ponderação T2, que evidenciam a lesão de HS. Cortes coronal, sagital e axial.

Já foi comprovado que há diferença na aferição do defeito nas imagens em duas ou três dimensões. A TC com reconstrução tridimensional (TC-3D) é o padrão-ouro para a quantificação do defeito de HS. 7 17 A aferição é feita por meio de cortes perpendiculares ao defeito ósseo, mas também apresenta variabilidade. 18 19

**Fig. 5:**
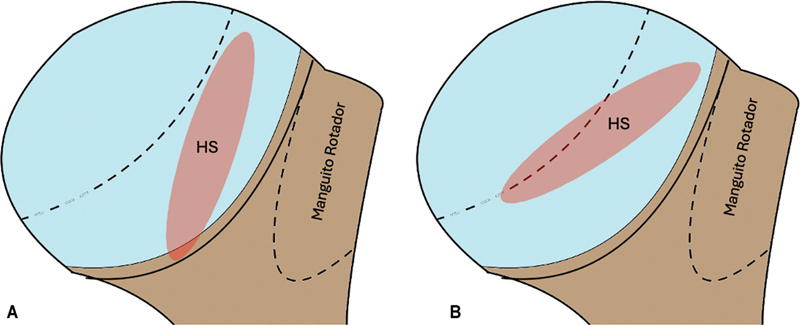
( **A** ) O ângulo é menor, e apresenta menor risco de *engagement* . ( **B** ) O ângulo é maior, e apresenta maior risco de *engagement* . Consideramos que, tanto em **A** quanto em **B** , o tamanho da lesão de HS seja o mesmo.

A angulação da lesão de HS é definida na TC-3D, entre a linha que passa profundamente ao vale da lesão de HS e o eixo longitudinal da diáfise umeral, conforme demonstrado na Fig. 5 . 17 Quanto maior essa angulação, maior é o risco de *engagement* .

**Fig. 6:**
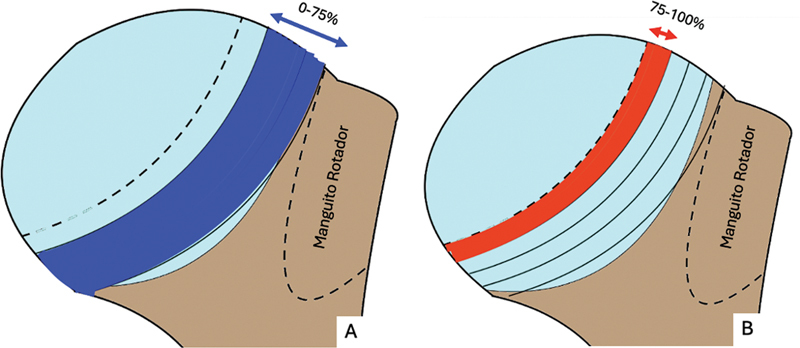
Imagem de RM aferida no corte axial (seta preta); imagem de TC tridimensional aferida perpendicularmente ao defeito (seta azul). Deve-se observar que o tamanho da seta preta é maior do que o da azul, o que pode acarretar a superestimação do defeito e alterar a conduta do caso.

Atualmente, o uso da RM com reconstrução tridimensional (RM-3D) vem aumentando. Estudos 20 21 relataram equivalência na acurácia da aferição do defeito da lesão de HS entre a TC-3D e a RM-3D. A RM-3D apresenta a vantagem de não utilizar radiação ionizante, além de oferecer maior potencial diagnóstico para lesões em partes moles, como na avaliação da inserção do manguito rotador. Já na TC-3D, essa avaliação pode resultar em alta variabilidade e baixa concordância interobservador, devido à dificuldade de visualização dessa estrutura. Entre as desvantagens dos métodos de reconstrução estão o alto custo e a limitada acessibilidade. É importante destacar que, ao aferir o tamanho da lesão de HS por meio de TC ou RM bidimensional, é mais provável obter uma medida superestimada (realizada na diagonal), o que aumenta a chance de que a lesão seja classificada como *off-track* e pode influenciar a escolha da técnica cirúrgica a ser utilizada ( Fig. 6 ).

## Diagnósticos Diferenciais

Ao associar o histórico clínico do paciente aos exames de imagem, torna-se mais fácil diagnosticar as lesões de HS. No entanto, é importante lembrar que algumas condições podem causar erosões ósseas na cabeça umeral, simulando essas lesões, como espondilite anquilosante, artrite reumatoide, artrite séptica, hiperparatireoidismo, doença de depósito de hidroxiapatita, tumor maligno ou cistos benignos. 14

## Classificação

A lesão de HS pode ser classificada segundo os critérios de visualização artroscópica descritos por Calandra, 7 e baseia-se na quantificação da profundidade do defeito. O grau 1 é confinado à cartilagem articular, o grau 2 se estende até o osso subcondral, e o grau 3 apresenta um grande defeito subcondral. 16 No entanto, sua aplicabilidade clínica é limitada.

**Fig. 7:**
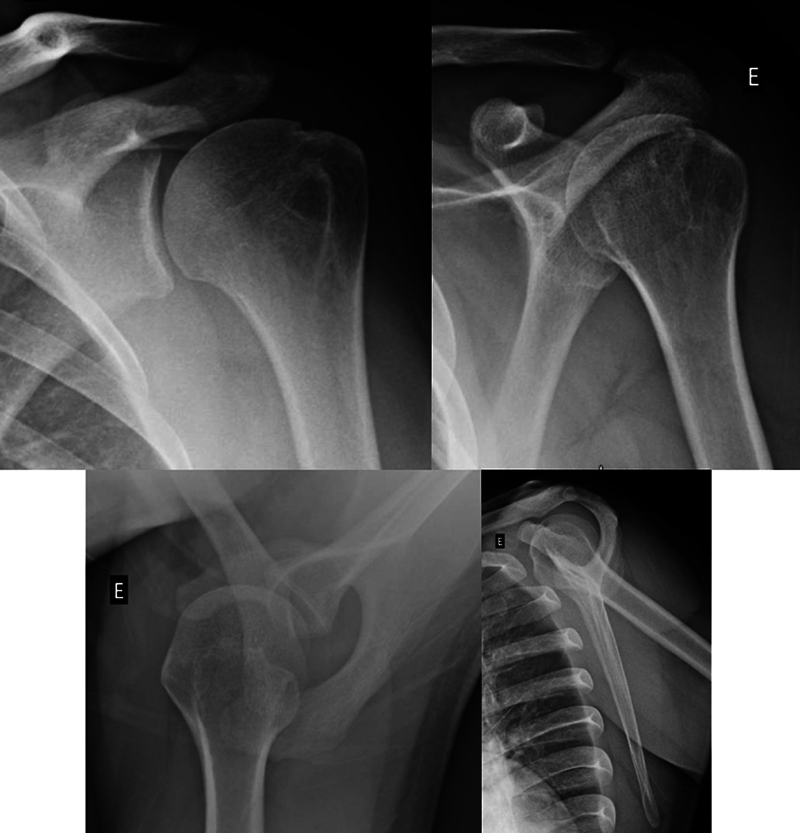
A seta indica a mudança da região de contato do úmero com a borda da cavidade glenoidal na posição de repouso para a posição de abdução e rotação externa (ABRE).

Um dos conceitos mais importantes relacionados às lesões de HS é o *glenoid track* (GT), que é a área de contato entre a cavidade glenoidal e a cabeça umeral durante o movimento, desde a posição neutra até a posição de abdução e rotação externa (ABRE). 20 Nesse movimento, a área de contato se desloca de inferomedial para superolateral na cabeça umeral ( Fig. 7 ). Estudos cadavéricos mostram que a área coberta pela cavidade glenoidal corresponde a 84%, enquanto estudos clínicos indicam uma cobertura de 83%. 19

Podemos usar o lado contralateral da cavidade glenoidal para avaliar o tamanho do defeito ósseo; no entanto, é necessário cautela, pois em 8% das cavidades glenoidais existe uma diferença ≥ 3 mm em relação ao lado contralateral.

O método de Di Giacomo et al. 9 para a aferição do GT consiste em 4 passos:

1. Aferição do diâmetro da cavidade glenoidal inferior usando um círculo perfeito (D);
2. Aferição da perda óssea anterior da cavidade glenoidal (d);
3. Cálculo da largura do GT = (0,83 × D) − d
4. Aferição da largura do intervalo de Hill-Sachs (IHS) = largura do HS + largura da ponte óssea (PO).

Se o IHS > GT, considera-se a lesão de HS *off-track,* ou seja, com risco de *engagement.*

**Fig. 8:**
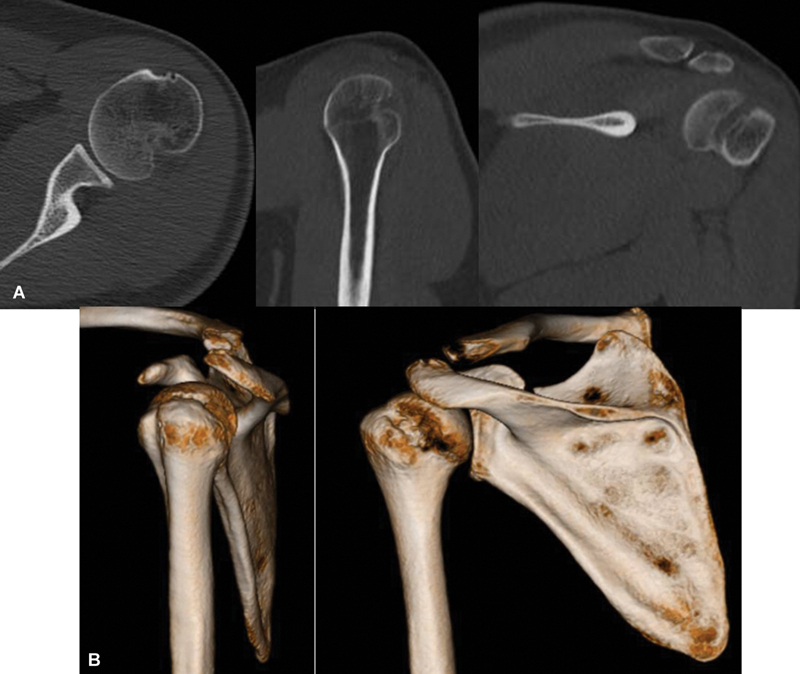
( **A** ) Lesão *on-track* (intervalo de Hill-Sachs [IHS] < *glenoid track* [GT]); ( **B** ) lesão *off-track* (IHS > GT).

Se o GT > IHS, considera-se a lesão de HS *on-track* , ou seja, sem risco de *engagement* 17 ( Fig. 8 ).

Apesar de ser um método frequentemente utilizado na prática clínica, as concordâncias intra e interobservador são baixas, principalmente devido à dificuldade em definir a margem medial da lesão de HS, a inserção correta do manguito rotador na TC, e a sobreposição da borda lateral da lesão de HS à borda medial da inserção do manguito rotador. 18 19 Outra crítica ao método do GT é que ele não considera a mobilidade articular, especialmente em indivíduos com frouxidão ligamentar, o que pode resultar em uma maior excursão da cabeça umeral. 21

**Fig. 9:**
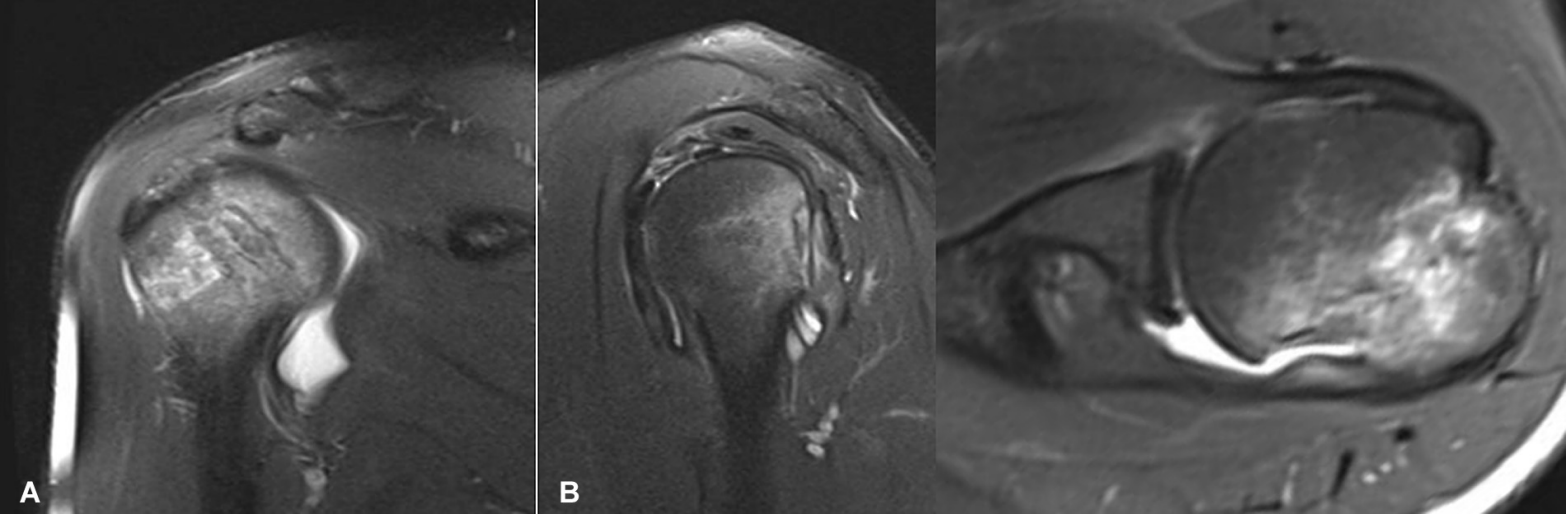
( **A** ) *Track* central (0% a 75% do GT); ( **B** ) *track* periférico (75–100% do GT).

Outra classificação por método de imagem que pode ser utilizada é a da localização do defeito de HS, desenvolvida a partir da observação de que até mesmo algumas lesões *on-track* submetidas à cirurgia de Bankart isoladamente evoluíram com falha. Essa classificação divide o GT da cabeça umeral em quatro zonas, e demonstrou-se 19 que as lesões de HS que atingem a zona periférica mais medial ( *track* periférico) apresentam piores resultados segundo o Western Ontario Shoulder Instability Index (WOSI) quando comparadas às demais zonas ( *track* central) **(** Fig. 9 ).

Outros métodos de aferição vêm sendo desenvolvidos para diminuir as falhas em lesões consideradas *on-track* tratadas apenas com reparo do lábio. O *distance to dislocation* (DTD) 22 considera a extensão inferior craniocaudal da lesão de HS. 5 Outro método é o *global track* , 21 que utiliza o domo da cabeça umeral e seu ponto central como referência, em vez da inserção do manguito rotador, para a aferição do defeito de HS. No entanto, esses métodos ainda precisam ser validados e testados na prática clínica.

## Tratamento

O tratamento da instabilidade anterior do ombro, a que as lesões de HS estão frequentemente associadas, pode ser realizado de maneira não operatória em pacientes com baixa demanda no primeiro episódio de luxação. Esse tratamento consiste no fortalecimento do deltoide, do manguito rotador e dos estabilizadores da escápula. É importante estar atento aos fatores de risco de recorrência, como a idade (menor do que 20 a 25 anos), o sexo masculino, a epilepsia, o risco de queda, a frouxidão ligamentar e a participação em atividades que requerem ABRE ou em esportes competitivos. 7 23 24

Nos casos em que há indicação de cirurgia, o tratamento da lesão de HS não é habitualmente realizado de maneira isolada, uma vez que a lesão labial ou do complexo ligamentar quase sempre está presente, e sua associação com o defeito ósseo da cavidade glenoidal (lesão bipolar) ocorre em 63% dos casos. 7

A presença da lesão de HS é fundamental para a decisão terapêutica, uma vez que o seu tamanho, localização e inclinação influenciam no tratamento. Alguns autores 7 25 defendem que, nos casos com defeito ósseo pequeno (< 20% da cabeça umeral) ou na ausência de *engagement* , pode-se realizar o reparo de labial isolado. Nos casos com defeitos maiores ou na presença de *engagement* , devemos considerar outros procedimentos. Devido ao fato de ocorrerem alguns casos de falha, até mesmo na ausência *engagement* , há uma crescente tendência de que a lesão de HS seja abordada nos casos de instabilidade anterior, inclusive nas situações com lesões ósseas limítrofes, especialmente com a técnica de *remplissage* . 21 22 26

## Procedimentos na Cabeça Umeral

A técnica de *remplissage* foi descrita em 1972, e vem do francês “preenchimento”. Foi modificada por Wolf para a realização por artroscopia, na qual se faz o “preenchimento” da lesão de HS pela capsulodese combinada com a tenodese do infraespinal, sempre associada ao reparo labial. A prevalência das lesões *off-track* gira em torno de 7%. 27 O sucesso dessa técnica é determinado pela habilidade de converter a lesão de HS de intra-articular para extra-articular, o que reduz o *engagement* com a borda inferior da cavidade glenoidal e a subluxação recorrente. Atualmente, a taxa de recidiva varia entre 0% e 10%; 28 29 contudo, o tamanho do defeito ósseo da cavidade glenoidal tem influência nestes valores.

A vantagem desse procedimento é a abordagem minimamente invasiva, que evita a necessidade de cirurgias de bloqueio ósseo da cavidade glenoidal, assim como suas complicações. A desvantagem teórica dessa técnica é a alteração da anatomia do manguito rotador e da biomecânica do ombro, que pode reduzir a rotação lateral e gerar dor na região posterossuperior do ombro. 30 Todavia, este é um tópico controverso, com alguns estudos não demonstrando diferença nesses desfechos em comparação à técnica isolada de reparo labial, principalmente com o desenvolvimento da técnica e estudos sobre o local ideal da inserção das âncoras.

Há outras opções na abordagem da lesão de HS que consistem em dois tipos de procedimento: umeroplastia (utilizado para defeitos agudos) e enxerto ósseo osteocondral.

A umeroplastia (elevação da fratura impactada e apoio com enxerto) restaura a geometria da cabeça umeral sem fixação interna. Ela é indicada para lesões agudas de até 3 semanas, que apresentam menos do que 40% de acometimento da superfície articular. 3

Esse método consiste na elevação da cartilagem e no preenchimento do defeito com fosfato de cálcio, 31 o que restaura a anatomia local e proporciona ganho de 5° na rotação lateral. Outra maneira de se elevar o defeito é com um balão de vertebroplastia de forma percutânea, com possibilidade de assistência por videoartroscopia, 32 33 com relatório demonstrando redução de 99,3% no defeito ósseo da lesão HS. 7

A artroplastia parcial com o uso de aloenxerto consiste no preenchimento do defeito com enxerto osteocondral pelas vias aberta ou artroscópica. O uso do aloenxerto apresenta a vantagem de restaurar melhor a biomecânica, ao contrário dos enxertos não anatômicos. As possíveis desvantagens são a possibilidade de transmissão de doenças, a dificuldade do procedimento, a reabsorção ou a falha do enxerto, a subluxação e a formação de cistos. 7 Também pode haver uma diferença entre o tamanho das geometrias do defeito e do implante, sendo necessária uma fresagem da cartilagem do úmero.

## Procedimentos da Cavidade Glenoidal

O tratamento da lesão da cavidade glenoidal depende do tamanho do defeito ósseo. Atualmente, o reparo de Bankart isoladamente apresenta indicações restritas. A literatura 34 vem apresentando uma perspectiva de prognóstico cada vez mais reservado em relação à perda óssea crítica para a realização do reparo de Bankart. Ao adicionar o *remplissage* ao reparo de Bankart, é possível reduzir o número de falhas do tratamento cirúrgico. 23 35

Os procedimentos ósseos aumentam a superfície da cavidade glenoidal e tratam indiretamente potenciais falhas que a lesão de HS poderia ocasionar. As técnicas mais utilizadas e difundidas são o procedimento de Latarjet, a técnica de Éden-Hybinette e o uso de aloenxerto de tíbia.

As possíveis desvantagens da técnica com enxertos ósseos na cavidade glenoidal são a perda de rotação lateral (média de 11°), infecção, hematoma, reabsorção do enxerto, pseudoartrose ou união fibrosa e lesão do subescapular. 23 30

## Artroplastias

As próteses parciais de recobrimento ( *resurfacing* ) apresentam como vantagens a ausência de morbidade do sítio doador em comparação com autoenxerto e o menor tempo cirúrgico. As desvantagens são a dificuldade de se obter fixação adequada e a incapacidade de se alinhar a superfície da prótese com a superfície articular do úmero. 36

Outra opção é a hemiartroplastia, procedimento indicado para pacientes com idade avançada e com defeito de HS > 40% da superfície articular. Para aumentar a estabilidade, podemos incrementar a retroversão em 10° a 15°. 7 25

Outros tipos de artroplastia são pouco frequentes e reservados às instabilidades mais graves ou a casos de complicações após procedimentos prévios.

## Opções de Interesse Histórico

A osteotomia derrotativa de Weber é uma opção de tratamento, mas ainda hoje pode ser utilizada como procedimento de salvação (em pacientes jovens). Nessa técnica, realiza-se uma osteotomia no colo cirúrgico do úmero e uma retroversão da cabeça umeral em relação à diáfise. Trata-se de um procedimento com resultados variáveis e índices de complicações relativamente altos, como pseudartrose, fratura iatrogênica e artrose. 7

## Considerações Finais

A lesão de HS tem papel fundamental na instabilidade do ombro e, recentemente, a pesquisa sobre este assunto tem aumentado de modo exponencial.

Tem-se buscado um algoritmo para a tomada de decisão sobre o melhor tratamento dos pacientes com instabilidade anterior do ombro, mas, apesar de todas as tentativas e os conceitos desenvolvidos, até o momento não existe um algoritmo ideal.

Assim, para a melhora dos resultados com taxas altas de sucesso, sem recidiva da luxação e com ótima função, levando-se em conta as expectativas dos pacientes, é fundamental considerar as lesões de HS e seu correto tratamento. Ainda se espera que essa evolução que estamos testemunhando traga melhores resultados funcionais e menos complicações de forma duradoura a esses pacientes que, como já mencionado, são em sua maioria jovens ativos e com altas expectativas.
